# Improving the Annotation of *Arabidopsis lyrata* Using RNA-Seq Data

**DOI:** 10.1371/journal.pone.0137391

**Published:** 2015-09-18

**Authors:** Vimal Rawat, Ahmed Abdelsamad, Björn Pietzenuk, Danelle K. Seymour, Daniel Koenig, Detlef Weigel, Ales Pecinka, Korbinian Schneeberger

**Affiliations:** 1 Department of Plant Developmental Biology, Max Planck Institute for Plant Breeding Research, Carl-von-Linné-Weg 10, 50829, Cologne, Germany; 2 Department of Plant Breeding and Genetics, Max Planck Institute for Plant Breeding Research, Carl-von-Linné-Weg 10, 50829, Cologne, Germany; 3 Department of Genetics, Cairo University, 12613, Giza, Egypt; 4 Department for Molecular Biology, Max Planck Institute for Developmental Biology, Spemannstrasse 35–39, 72076, Tübingen, Germany; University of Toronto, CANADA

## Abstract

Gene model annotations are important community resources that ensure comparability and reproducibility of analyses and are typically the first step for functional annotation of genomic regions. Without up-to-date genome annotations, genome sequences cannot be used to maximum advantage. It is therefore essential to regularly update gene annotations by integrating the latest information to guarantee that reference annotations can remain a common basis for various types of analyses. Here, we report an improvement of the *Arabidopsis lyrata* gene annotation using extensive RNA-seq data. This new annotation consists of 31,132 protein coding gene models in addition to 2,089 genes with high similarity to transposable elements. Overall, ~87% of the gene models are corroborated by evidence of expression and 2,235 of these models feature multiple transcripts. Our updated gene annotation corrects hundreds of incorrectly split or merged gene models in the original annotation, and as a result the identification of alternative splicing events and differential isoform usage are vastly improved.

## Introduction

*Arabidopsis lyrata* is a predominantly self-incompatible, perennial plant species that diverged from a common ancestor with *A*. *thaliana* approximately 10 million years ago [1]. Despite its evolutionary closeness, its genome size is estimated to be between 230 to 245 Mb, or one and a half times as large as the *A*. *thaliana* genome [2,3]. Except for *A*. *thaliana*, *A*. *lyrata* is the only species within the family of Brassicaceae with a reference assembly exclusively based on high quality dideoxy sequencing. This 207 Mb *A*. *lyrata* reference assembly attributed the difference in genome size between the two species to the accumulation of many small deletions in the *A*. *thaliana* genome primarily in non-coding regions and transposable elements (TEs) [1]. In addition, *A*. *lyrata* has experienced recent genome expansion due to activity of TEs, in particular Copia long terminal repeat (LTR) retrotransposons [1,4,5], which is the basis for species-specific patterns in DNA methylation [6].

As *A*. *lyrata* is the closest fully assembled relative of *A*. *thaliana*, it serves as an important out-group for evolutionary studies within *A*. *thaliana* [7–9]. Moreover, recent advances in sequencing technology have enabled the assembly of an increasing number of Brassicaceae genomes and their close relatives [4,5,10–19], which, together, are leveraged for comparative genomics in this family. Intra- as well as inter-species comparisons, however, heavily rely on the gene annotations of each species involved and high quality annotations even in the non-model species become essential.

Methods for gene model annotation profited considerably from the invention of high-throughput RNA sequencing (RNA-seq) [20,21]. Identification of genuine transcription start and termination sites as well as intron/exon borders is a non-trivial task when using only reference sequences and homology data. Now, information on spliced alignments from RNA-seq data can improve the identification of gene models [22,23] and also enable the annotation of variant isoforms [24]. In particular, the gene annotations of model species have been updated regularly despite only minor changes to the reference genome sequence [25].

The current gene annotation of the *A*. *lyrata* includes 32,670 genes and was generated using a combination of *ab initio* gene prediction, homology to known proteins, as well as gene sequences and expression data from related species [1]. Even though the gene models were analyzed for their expression support using RNA-seq data, gene prediction methods integrating RNA-seq alignment information had not been developed at the time the assembly had been generated. In a recent study, Haudry and colleagues supplemented the original annotation with additional putatively transcribed regions in order to study the conservation of non-coding sequences among related Brassicaceae species [14]. They integrated the results of additional *ab initio* gene predictions, RNA-seq data alignments and homology searches against the genes of *A*. *thaliana* in order to mask potentially un-annotated coding sequences and regions that recently lost coding potential due to mutations.

Building upon the major efforts of the first annotation of *A*. *lyrata* genome (version-1 from hereon) we have updated the gene models using diverse RNA-seq samples. Our annotation (version-2 from hereon) has changed the coordinates of 29,141 of the original 32,670 gene models, removed 1,286 and added 1,295 new models. This update corrected hundreds of gene models, which were wrongly merged or split in version-1, and also separated transposable element genes from other protein coding genes. Finally, we have analyzed the transcriptional response of *A*. *lyrata* to heat stress to show the improved utility of version-2 for the identification of differential isoform usage and pre-mRNA splicing.

## Results and Discussion

### Improving the *A*. *lyrata* gene annotation using transcriptional data

We sequenced the transcriptome of various *A*. *lyrata* aerial tissues, including whole rosettes, dissected shoot apices, complete inflorescences, as well as vegetative rosettes exposed to cold and heat stress (see Materials and Methods). In total, we generated over 290 million single-end, strand unspecific short reads using Illumina sequencing technology after poly-A purification (Table 1). Short reads were aligned to the *A*. *lyrata* reference assembly [1] using Bowtie v2.1.0 [26] and the splice junction mapper TopHat v2.0.9 [27] (see Materials and Methods). We could align 89% of all reads, out of which 85% aligned uniquely and were used for further analysis. The proportion of unaligned reads was comparable to the proportion of unaligned reads in similar experiments with *A*. *thaliana*, which presumably has one of the most complete reference genome sequences. Over 10% of the aligned reads matched to putative intergenic regions indicating that some gene models may have been missed in the original version of the *A*. *lyrata* gene annotation. Visual inspection of these intergenic alignments revealed the expected patterns for spliced transcripts indicating instances of unidentified gene models and cases where transcription exceeded known gene boundaries (see Fig A in S1 Dataset).

**Table 1:** Short read statistics (read numbers in millions).

Tissue	Sample	Read length (bp)	Raw reads	Reads aligned	Reads aligned uniquely (full-length alignments)	Reads aligned uniquely (spliced alignments)
Rosette (WT)	Rep 1	96	16.0	11.9	8.3	3.0
Rosette (WT)	Rep 2	96	12.0	9.1	6.0	2.7
Rosette (Heat stressed)	Rep 1	96	17.6	13.1	9.7	3.4
Rosette (Heat stressed)	Rep 2	96	5.9	4.6	3.1	1.2
Rosette (Recovered)	Rep 1	96	15.6	12.6	5.6	4.1
Rosette (Recovered)	Rep 2	96	5.8	4.5	2.8	1.3
Shoot apical meristem (WT)	Rep 1	101	14.5	12.7	7.5	3.6
Rosette (WT)	Rep 1	101	19.6	17.5	9.3	5.6
Rosette (WT)	Rep 2	101	18.1	16.2	9.3	4.8
Inflorescence (WT)	Rep 1	75	32.0	30.2	12.9	9.3
Inflorescence (WT)	Rep 2	75	32.0	29.5	12.6	9.0
Rosette (WT and cold stressed)	Rep 1	75–100	102.7	96.7	59.7	24.6
			291.8	258.6	146.8	72.6

New gene models were predicted from short read alignment data using Cufflinks 2.1.1 [22] independently for each tissue. In total, Cufflinks predicted 31,194 distinct gene models across all samples. An additional RNA-seq alignment-guided gene prediction using Augustus v.3.0.1 [28] identified 40,728 gene models, including 27,830 genes, which were supported by at least five RNA-seq reads. Moreover, 30,483 and 30,837 of Augustus predicted gene models overlapped with version-1 and Cufflinks predictions, respectively (see Materials and Methods and Fig B in S1 Dataset).

**Fig 1:**
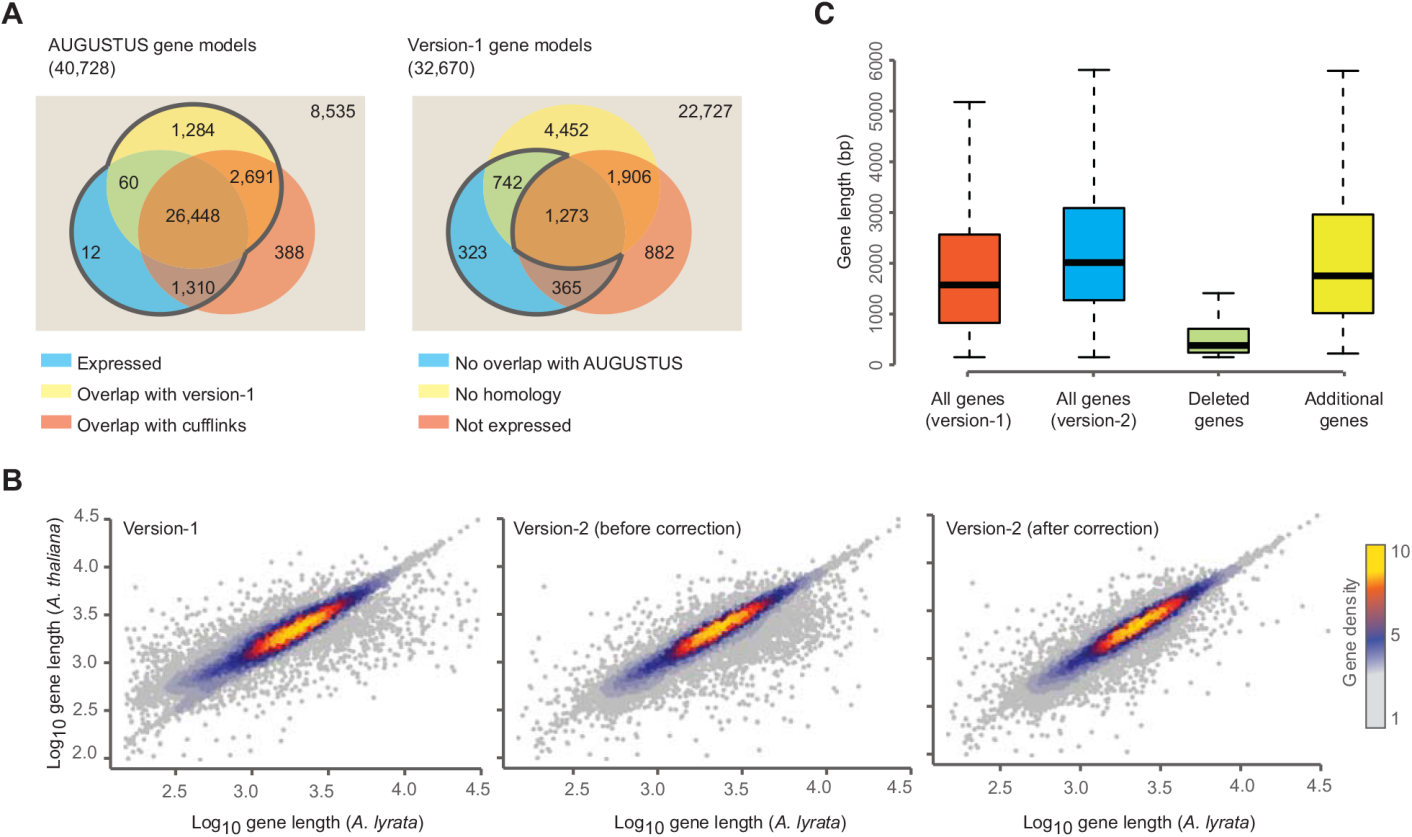
Updating the gene model annotation of *A*. *lyrata*. **(A)** Left, version-2 gene models predicted by Augustus [28]. Number of gene models overlapping with version-1 (yellow), genes predicted with Cufflinks (red), and genes with expression evidence (blue). Right, gene models of the version-1 annotation. Number of models without overlap to version-2 models (yellow), without orthologs in five other Brassicaceae (red), and without significant expression evidence (blue). **(B)** Correlation of the lengths of *A*. *lyrata* gene models with the length of their orthologous gene models in *A*. *thaliana*. Left, *A*. *lyrata* version-1 gene models. Correlations using version-1 gene models (left), version-2 gene models before (middle) and after (right) the homology-based correction of gene models. **(C)** Length distribution of gene models including genes that were removed or newly added in the version-2.

We combined 31,793 Augustus gene models with evidence of transcription or that overlapped with version-1 gene models to update the *A*. *lyrata* gene annotation (Fig 1A). To ensure that we were not excluding any true gene models in version-1, we included 1,430 version-1 gene models that were not overlapping with any of the new gene models, but showed either evidence of expression or featured an ortholog in at least one of the Brassicaceae species *A*. *thaliana* [29], *Capsella rubella* [4], *Brassica rapa* [10], *Schrenkiella parvula* [11] and *Arabis alpina* [5]. This increased the number of gene models to 33,223 (see Materials and Methods). To identify and to correct cases where incorrect gene models may have been introduced into the version-2 annotation, we utilized the very close phylogenetic relationship between *A*. *lyrata*, *A*. *thaliana* and *C*. *rubella*. We compared all gene models that were considerably different between version-1 and version-2 to *A*. *thaliana* and *C*. *rubella* orthologs (see Materials and Methods). If the length of the version-1 open reading frame was closer to that of the orthologs, we retained the version-1 gene model. This resulted in 548 version-2 gene models being replaced with 688 of the original version-1 gene models (Fig 1B). After additional removal of redundant gene models we obtained a final set of 33,221 non-redundant gene models.

Based on a recent annotation of *A*. *lyrata* TEs [14] and sequence similarity to TE genes of *A. thaliana [25]*, we annotated 2,089 of the protein coding gene models as TE protein coding genes (see Materials and Methods). Without these, version-2 comprised of 31,132 gene models, which is ~13% more than in *A*. *thaliana* [25]. Although tRNA genes were described in the original analysis of the *A*. *lyrata* genome [1], version-1 lacks information regarding these loci. By rerunning tRNAScan [30], we identified 660 tRNA genes coding for all 20 amino acids. For completeness, we also incorporated 170 recently published miRNA genes into the new annotation file [31].

Altogether, we updated the coordinates of 29,141 of the original gene models, removed 1,286 entire (mostly short) gene models, and added 1,295 new models (Fig 1C). Only 2,243 remained unaltered (including 688 version-1 gene models re-introduced due to their superior similarity to orthologs). The new annotation accounted for 31,132 non-TE related gene models including 27,084 multi-exonic genes of which 2,236 featured at least one alternative isoform (Table 2).

**Table 2:** Comparison of version-1 and version-2 annotations.

	# version-1	# version-2
Gene models	32,670	33,221
Predicted transcripts	32,670	35,805
Protein coding genes	32,670	31,132
TE coding genes	-	2,089
miRNA genes	-	170
tRNA genes	-	660
Featuring ortholog (in at least one Brassicaceae)	23,996	24,146

### Validating differences in gene model structure

**Fig 2:**
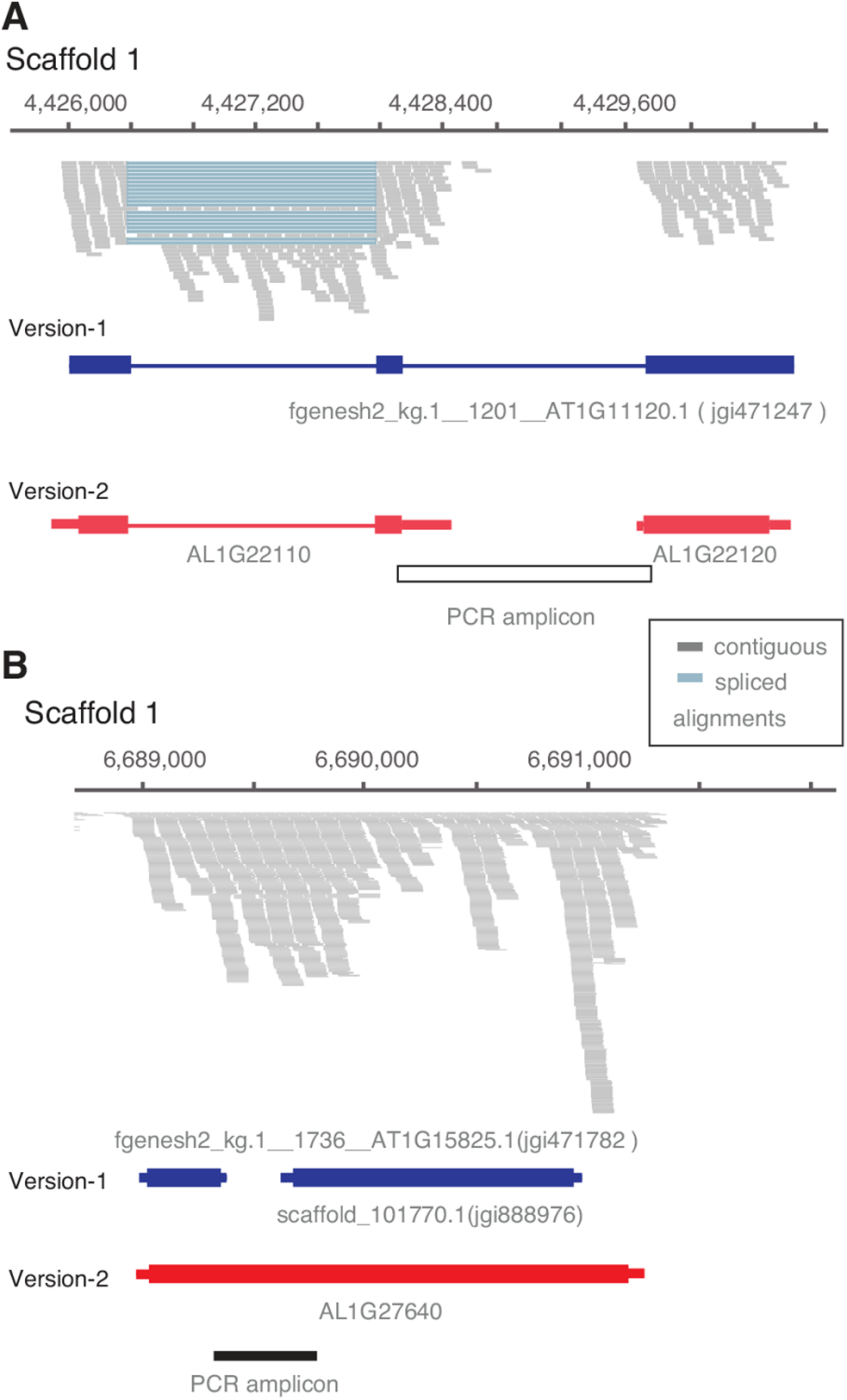
Examples of version-1 gene models split and merged in *A*. *lyrata* gene annotation version-2. **(A)** Example of a gene model that was split into two gene models in version-2. Reverse transcription-PCR could not confirm the connection of both. **(B)** Example of version-1 gene models that were merged during the annotation update. Reverse transcription-PCR confirmed presence of a transcript bridging the two version-1 genes.

Even after the above-mentioned homology-based gene length adjustments, we found cases where the corresponding gene models from the two annotations varied drastically in length. This included instances where multiple version-1 gene models were fused to form a single gene model in version-2 or vice versa (Fig 2). In total, 161 version-1 genes were split (accounting for 530 genes in version-2) and 1,729 version-1 gene models were merged (accounting for 775 gene models in version-2). We randomly selected 14 version-1 gene models that had been split into multiple gene models in version-2, and another 14 gene models that had been merged in version-2, for PCR validation (see Fig C and D in S1 Dataset). One split case did not yield gDNA bands indicating a technical problem in primer design. For three merge cases we obtained cDNA bands of the expected size, but were not able to amplify genomic DNA for primer validation. This was most likely due to large gDNA amplicon size (2.4–5 kbp) and rendered the results of these cases inconclusive. For all 24 remaining cases, PCR results fully confirmed the annotation of the new gene models.

### *A*. *lyrata* version-2 annotation in contrast to other Brassicaceae annotations

**Fig 3:**
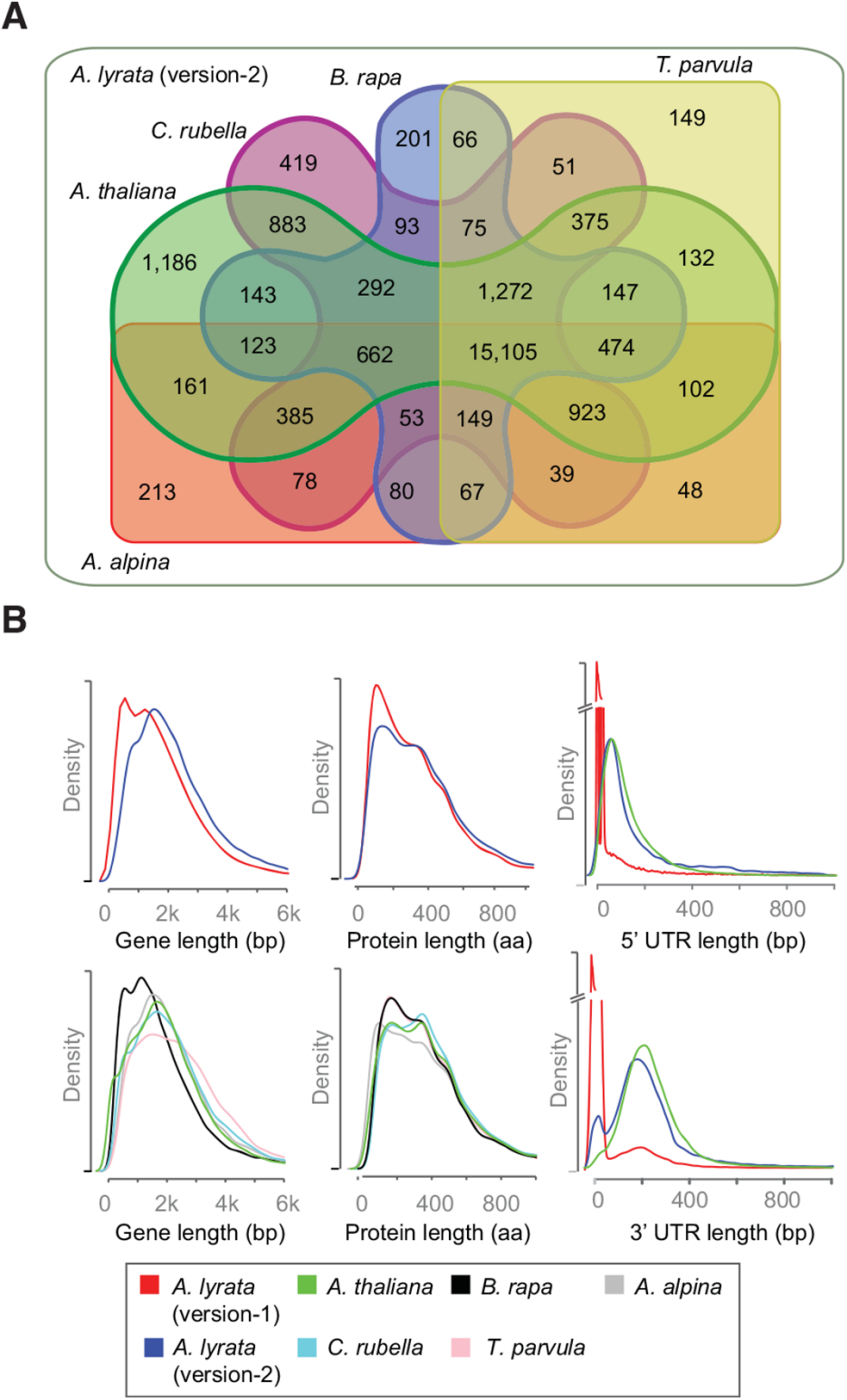
Comparing the *A*. *lyrata* gene annotation version-2 with the annotations of five other Brassicaceae. **(A)** Orthologous gene models shared between *A*. *lyrata* (version-2), *A*. *thaliana* [29], *A*. *alpina* [5], *B*. *rapa* [10], *C*. *rubella* [4] and *S*. *parvula* [11]. **(B)** Gene, Protein and UTR length distributions of above-mentioned species including the new and old *A*. *lyrata* annotations. UTR distribution is only shown for *A*. *lyrata* and *A*. *thaliana* because of poor UTR annotation in some of the other species.

For both *A*. *lyrata* annotations we predicted orthologous relationships between *A*. *lyrata* and five other Brassicaceae species (see Materials and Methods). Using version-2 gene models, 77.5% of genes had an ortholog in at least one species (24,146 out of 31,132) (Fig 3A), compared to 73% for version-1 (23,996 out of 32,670) (see Fig E in S1 Dataset). The number of genes with orthologs in all five Brassicaceae was also slightly higher for version-2 with 15,105 genes versus 14,850 genes with version-1.

The removal of many short gene models in version-2 changed the distribution of gene model lengths (Figs 1C and 3B). Version-1 has an excess of gene models shorter than 1 kb with a second mode around 1.5 kb, which describes a bimodal distribution that was only reflected by gene length distribution of *B*. *rapa* In contrast version-2 had only a single mode around 1.7 kb, similar to the four other species. The length distribution of predicted protein sequences in version-1 had also been distinct from the other Brassicaceae species, and this discrepancy largely disappeared with version-2. A third factor that contributed to the length differences between the genes of version-1 and version-2 were differences in UTR annotations (Fig 3B). In version-1 33% of the genes were annotated without UTR information, however, in version-2 only 5% remained witout 3’ and 5’ UTR annotation. The absolute and relative contributions of individual features are shown in Fig F in S1 Dataset. Though, absolute increase in genomic space for all gene features was observed but CDS and UTRs are benefited the most. We also observed little decrease in intronic genome space, which can be explained by introduction of splice variants previously missing from version-1 annotation.

Whether the bimodal distribution in *B*. *rapa* reflects similar ambiguity in gene annotations, or mirrors particular characteristics of *B*. *rapa*, including its ancient genome triplication and subsequent fractionation, is not known.

### New annotation enabled improved identification of alternative splicing events

The availability of multiple isoforms from individual gene models in version-2 enables quantitative expression comparisons between annotated isoforms. We analyzed RNA-seq data from *A*. *lyrata* rosette tissues from untreated (WT), heat stressed (HS), and recovered (REC) samples in duplicate (see Materials and Methods). We first analyzed the data for differential gene expression using Cuffdiff v.2.0.2 [32]. WT and REC differed from HS at 3,114 and 2,962 genes, whereas only 106 genes differed between WT and REC. This indicates, as expected, a strong effect of heat stress on gene expression (see Materials and Methods). Cuffdiff was also used to estimate differential expression between isoforms. We identified differential isoform expression at 283, 15 and 119 genes when comparing WT with HS, WT with REC, and HS with REC, respectively. In contrast, as version-1 does not include different isoforms, which are prerequisite for isoform expression analysis as implemented in Cuffdiff, it was not possible run this analysis using version-1.

**Fig 4:**
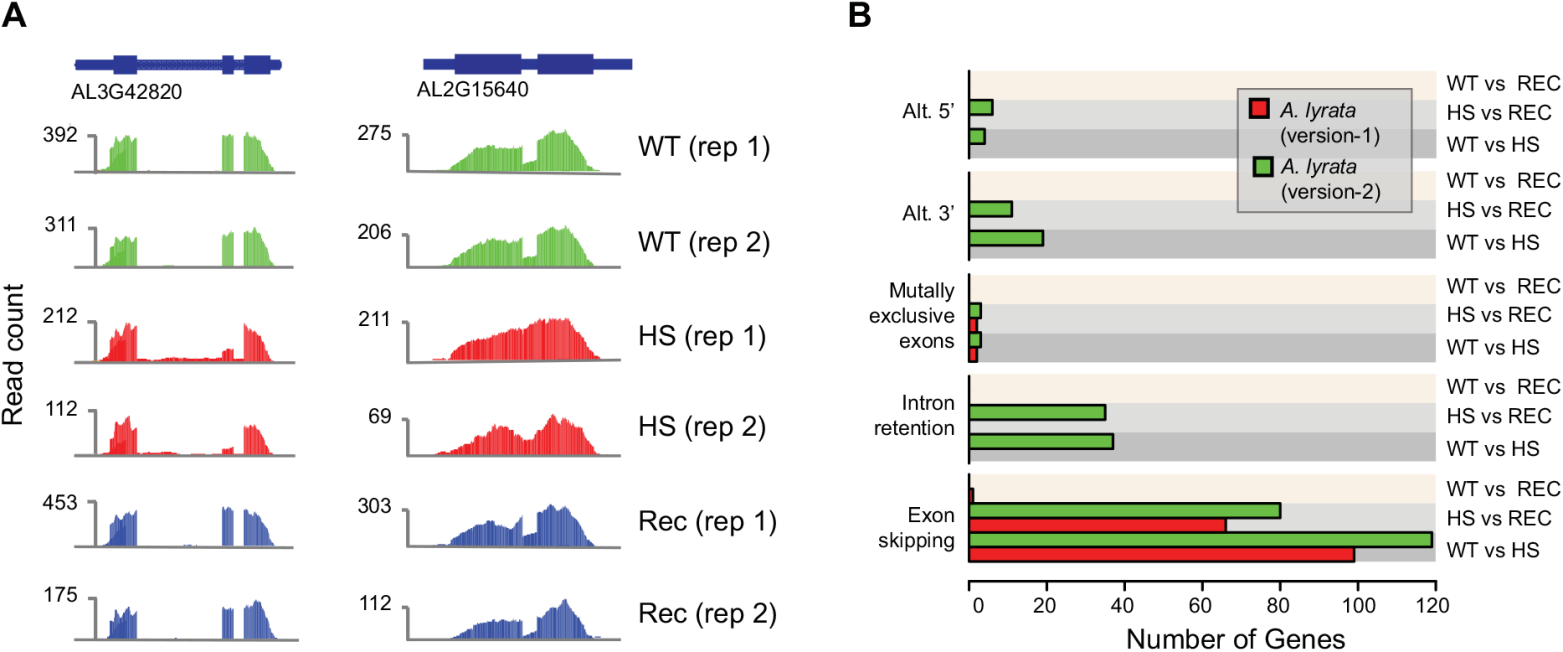
Heat stress induces alternative splicing events. **(A)** Examples of differentially expressed isoforms in response to heat stress in *A*. *lyrata*. AL3G42820 expresses a second isoform that lacks the middle exon in heat-treated samples (HS). Transcripts from wild-type (WT) and recovery (REC) samples contain all three exons. AL2G15640 retains an intron in response to heat stress (HS) while wild-type (WT) and recovery (REC) samples show partial intron splicing. **(B)** Number of differential splicing events, including alternative 5’ and 3’ splice sites, mutually exclusive exons, intron retention, and exon skipping events identified with MATs based on version-1 and version-2 annotations.

We investigated differential splicing using a second tool, MATS v3.0.8 [33], which does not rely on prior isoform annotations and only identifies differences in individual splicing events, but not between entire transcripts. With version-2, MATS identified 177, 0 and 130 differential splicing events distributed over 187 distinct gene models in the three comparisons (Fig 4; see Materials and Methods). MATS reported only 99, 1 and 67 events affecting 103 gene models using version-1. The overlap of different splicing events was very high (95 out of 103 (version-1) and 187 (version-2) gene models). Thus, almost all gene models with differential splicing events predicted based on version-1 were also predicted using version-2, however, the results based on version-2 revealed many more gene models. This was partially due to newly added genes (10 cases), but the most improvement came from the updates to exon-intron boundaries of existing gene models indicating that the new gene annotation improved the overall utility of this resource.

The isoform-dependent (Cuffdiff) and-independent (MATS) analyses identified only 37 common gene models. Even though Cuffdiff revealed fewer events as compared to the MATS analysis, it did identify 100 genes with differential isoform usage that were not included in the set of genes with multiple isoforms. This suggests that differential isoform expression analysis profits from prior isoform annotation, however, should not only rely on existing isoforms.

### Availability of the annotation and gene naming conventions

The version-2 annotation can be found in S2 Dataset. The gene identifiers have been updated following the annotation principles applied to *A*. *thaliana* [29]. Gene numbering follows the physical order of genes on chromosomes, where each gene is named “AL” followed by scaffold number, then a “G” (for the first 8 scaffolds corresponding to the eight chromosomes) or “U” (for unanchored scaffolds) and finally a unique number incremented by 10, to leave flexibility for genes that were missed in this annotation. Genes that were removed from version-1 can be found in S3 Dataset. A mapping of the gene model identifiers of version-1 to version-2 can be found in S4 Dataset.

## Conclusions

The updated annotation includes 31,132 gene models with 35,805 transcripts. We also reported 1,304 gene models that were erroneously split or merged in the previous annotation. Validation of these models strongly supported our updates highlighting the importance of employing species-specific RNA-seq data for annotating genomes.

We also provided a first annotation of alternative splicing events in *A*. *lyrata*. Using RNA-seq samples for a heat stress experiment we demonstrated the improved utility of the version-2 annotation for differential isoform expression studies. This revised genome annotation advances the reference sequence of *A*. *lyrata* as a community resource for comparative and functional studies.

## Material and Methods

### Plant material

*Arabidopsis lyrata* subsp. *lyrata* MN47 plants were grown in soil under long day conditions (16 hours light, 21°C: 8 hours dark, 16°C). Vegetative rosettes and dissected shoot apices of three week old plants and entire inflorescences of flowering plants were harvested as mock treated samples. For heat stress and recovery treatments we incubated three week old plants at 37°C for 6 hours or for an additional 48 hours at 21°C, respectively. Cold stressed samples were treated as described [6].

### Nucleic acids isolation and RNA-seq library preparation

DNA was isolated using Nucleon Phytopure kit (GE Healthcare). For total RNA isolation, samples were flash frozen in liquid nitrogen and used with Qiagen RNeasy® Plant Mini Kit, including an on-column DNase I digestion. Total RNA integrity was confirmed on the Agilent BioAnalyzer. Barcoded libraries were constructed using the Illumina TruSeq RNA kit with average of 1 *μ*g of total RNA as starting material. The manufacturer's protocol was precisely followed with one exception in the cold-treated samples where 12 PCR cycles were used instead of the recommended 15. The library quality was monitored on a Bioanalyzer 2100 (Agilent) and the libraries were sequenced as 100-bp single end reads using Illumina sequencing.

### RNA-seq read mapping and gene predictions

RNA-seq data was mapped to the *A*. *lyrata* reference genome assembly [1] using Bowtie v1.0.0 [26] and TopHat v2.0.10 [27]. Cufflinks v2.0.2 [22] was used for *de novo* transcript identification in all tissues separately. Cuffmerge (from the Cufflinks suite) was used to merge transcript annotation files obtained for three tissues separately. In addition, all short reads were aligned to the reference assembly of *A*. *lyrata* using BLAT v.34 [34] to generate an evidence file for guided gene prediction using Augustus v3.0.0. *A*. *lyrata* specific configuration file was generated using the version-1 annotation. To estimate agreement between Augustus and version-1 gene models, gene models with > = 30% overlap (in respect to the shorter gene model) were considered. Gene models supported by five or more RNA-seq reads were considered as expressed irrespective of gene length.

To identify cases where wrong gene models were introduced in version-2, we first compared version-2 proteins (23,181 comparable proteins) with corresponding version-1 proteins. A total of 1,037 proteins were identified as outliers, where protein length difference was outside the range of +/- standard deviation of the distribution of length differences. For these cases version-1 and version-2 protein sequences were further compared against the proteins of their orthologs in *A*. *thaliana* [29] and *C*. *rubella* [4]. If both orthologs were more similar in length to the protein of version-1, the respective version-2 gene model was replaced with version-1.

### Ortholog identification

Orthologous gene identification for both version-1 and version-2 was done separately at protein level using reciprocal best hits using blastall v2.2.25 [35] and an e-value cutoff 0.001 among five Brassicaceae species.

### Identification of TE genes in version-2

Version-2 gene models harboring complete TEs [14] within their coding regions or were entirely spanned by a TE were annotated as “TE coding genes”. In addition 3,909 *A*. *thaliana* TE genes [25] and TIGR Brassicaceae specific repeat database [36] were used to identify TE genes using blastn v2.2.25 [35].

### cDNA preparation and PCR

Plants were grown on soil under long day conditions until the five-leaf stage reached after approximately three weeks. cDNA samples were prepared from 1 *μ*g total RNA of mock-treated rosettes using RevertAid First Strand cDNA Synthesis Kit with oligo d(T) primers (Thermo Scientific). Reverse transcriptase minus samples were processed in the same way without enzyme addition. PCR reactions were done in an Eppendorf thermal cycler using a standard program and the products were visualized on agarose gels stained with ethidium bromide. The PCR primer sequences can be found in S5 Dataset.

### Differential gene expression and alternative splicing

Cufflinks [22] was used to calculate differential gene expression level (FPKM) with p-value < 0.01 and log2-fold change difference of more than 2. MATS [33] was used to investigate differential splicing events with over 0.01% splicing difference at a p-value < 0.01 and a false discovery rate of less than 1%. To control for false positives, genes with 10,000 fold or more expression difference were excluded.

## Supporting Information

S1 Dataset Supplementary figures. (DOCX)

S2 Dataset General feature formatted (GFF) file describing version-2 annotation. (ZIP)

S3 Dataset GFF file describing genes that were removed from version-1. (ZIP)

S4 Dataset Table describing the mapping of version-1 to version-2 gene models. (XLSX)

S5 Dataset Primer information for gene model validation. (XLSX)
