# Supplementary material for: Improving the Annotation of Arabidopsis lyrata Using RNA-Seq Data
Source: PLoS One. 2015 Sep 18;10(9):e0137391. doi: 10.1371/journal.pone.0137391 (PMC4575116; doi:10.1371/journal.pone.0137391)
Supplement: S1 Dataset — (DOCX) [file pone.0137391.s001.docx]

Improving the annotation of *Arabidopsis lyrata* using RNA-seq data

# Supplementary figures

Rawat *et al.*

#

## Fig A | Examples of incorrectly annotated gene models in version-1 of *A. lyrata* gene annotation.

**(A)** A gene model was entirely missing, but its locus shows clear evidence of transcription and splicing based on RNA-seq alignments. **(B)** The boundaries of two gene models do not include the full extent of the transcribed region. In the case of Al_scaffold_001_1048, an entire exon was missing in version-1.

## Fig B | Workflow for incorporating RNA-seq data into the version-2 annotation.

##

## Fig C | Validation of gene models merged in version-2 annotation by reverse transcription PCR.

**(A)** Schematic drawing of two gene models in version-1 that were merged into one in version-2. PCR primers were designed to span regions predicted as intergenic in version-1. gDNA, genomic DNA; cDNA, complementary DNA, RT-, reverse transcription reaction without reverse transcriptase. ** indicate cases where gDNA reaction did not work, most likely due to large amplicon size (2.4 – 5 kb). **(B)** Validation of version-2 gene model combining three version-1 gene models. The principle follows description in (A), except that both junctions are validated (A and B).

## Fig D | Validation of gene model splits in version-2 by reverse transcription PCR.

**(A)** The scheme shows version-1 gene models that were split in version-2. PCR amplicons A and B were designed to target cDNA sequences common to both annotations, while amplicon C spanned a region predicted as intergenic in version-2. gDNA, genomic DNA; cDNA, complementary DNA, RT-, reverse transcription reaction without reverse transcriptase. **(B)** Additional cases tested using strategy described in (A) where only amplicon C was tested.

##

## Fig E | Orthologs with five other Brassicaceae based on version-1.

## Fig F | Genome space comparison between version-1 and version-2 annotation.
